# Femtosecond laser microfabrication of a fully-integrated optofluidic device for 3D imaging flow cytometry

**DOI:** 10.1038/s41598-025-93118-x

**Published:** 2025-04-08

**Authors:** Federico Sala, Petra Paiè, Alessia Candeo, Francesco Ceccarelli, Roberto Osellame, Andrea Bassi, Francesca Bragheri

**Affiliations:** 1 Istituto di Fotonica e Nanotecnologie, Consiglio Nazionale delle Ricerche, Piazza Leonardo da Vinci, 32, Milan, 20133 Italy; 2 Dipartimento di Fisica, Politecnico di Milano, Piazza Leonardo da Vinci, 32, Milan, 20133 Italy

**Keywords:** Femtosecond laser micromachining, Imaging flow cytometry, Superresolution, Optoelectronic devices and components, Microscopy

## Abstract

In recent years imaging flow cytometry (IFC) is gaining increasing attention as it combines the characteristics of conventional flow cytometry with optical microscopy techniques, allowing for high-throughput, multi-parameter screening of single cell populations. In the field of biology, the always increasing demand for high content morphological and spatial information led to the development of systems for volumetric imaging. However, current 3D IFC systems are often limited by the incompatibility with available microfluidic devices or by instrumental complexity that might lead to optical misalignment or mechanical instabilities in day-by-day operation. To this end, here we demonstrate the importance of advancing the laser fabrication technique by reporting on a fully integrated optofluidic platform composed of a borosilicate glass chip encompassing reconfigurable integrated photonic circuits for patterned light generation, bonded to a fused silica glass chip incorporating cylindrical hollow lenses, for light-sheet illumination, perfectly aligned to a microchannel where the sample under investigation flows. The system is capable of high-resolution imaging flow cytometry by implementing structured light sheet microscopy in a heterogeneously integrated platform with unprecedented stability. All the components are realized by femtosecond laser irradiation followed by chemical etching. The extreme level of integration permitted by the advanced optimization of the laser fabrication technique allowed the reduction of the assembled components and the absence of moving parts, thus ensuring durable alignment as well as mechanical and thermal stability both in short and long-term operation of the device, for the automated fluorescence signal acquisition during the sample flow.

## Introduction

Flow cytometry is a powerful technique used in biology and medicine to rapidly analyze and quantify the characteristics of cell populations or particles suspended in a fluid. In a flow cytometer the sample is flown in a capillary and passes through a laser beam, allowing to collect the emitted fluorescent signal intensity, with which is possible to retrieve various properties and parameters on the sample population. The technique permits rapid analysis of a large number of cells, which is ideal for high throughput screening and analyses, for example for clinical diagnostics, drug development and personalized medicine. Recently, a growing interest in the understanding of the specificity of the heterogeneity within cell populations has called for the retrieval of single-cell information. As an example, different tumor cells show distinct morphological and phenotypic profiles and may exhibit different biological behaviors. However, when focusing on cellular population heterogeneity, also flow cytometry has some limitations therefore in recent years flow cytometry has been increasingly integrated with other technologies, as mass spectrometry, spectroscopy, and imaging techniques, to provide complementary information and a multi-omic approach for a deeper understanding of the sample under study. Imaging flow cytometry (IFC)^1,2^ is considered a powerful technique to obtain rapid and precise single-cell information from a large heterogeneous population. In fact, IFC adds to the high throughput of flow cytometry the ability to capture detailed images of individual cells or particles, enabling the rapid analysis of the sample morphology and fluorescence of all the single cells in the population. This is particularly useful for studying cellular viability, morphology, organelle structure, and spatial relationships like colocalization between different cellular structures or biomolecules, enabling a comprehensive phenotypic profiling and characterization of complex, heterogeneous cellular populations. This has also been possible thanks to the evolution of new methods for the extraction of quantitative data, image segmentation, feature extraction, and statistical analysis of cellular properties^3^. Significant advancements in speed, sensitivity, and resolution have improved imaging flow cytometry. These improvements result from combining advanced microscopy techniques with microfluidics strategies^4–9^. However, these examples still suffer from the intrinsic compromise between throughput and image resolution, therefore high-throughput measurements are demonstrated only on 2D images and at the expenses of image quality. In fluorescence microscopy, peculiar illumination schemes can be exploited, allowing the whole 3D image reconstruction possibly at resolution overcoming the diffraction limit. However, the introduction of high spatial content imaging techniques in flow cytometry typically limits the throughput of the analysis. The IFC technology has also been implemented commercially but still shows some drawbacks and challenges as it typically entails long experimental times due to the added step of imaging individual cells with respect to standard flow cytomery. Moreover, instruments are complex and delicate, due to the combination of multiple optical and imaging components, requiring precise alignment and regular calibration for accurate results, increasing the risk of mechanical failures, optical misalignments, and electronic malfunctions, demanding diligent maintenance, and troubleshooting efforts which can be challenging and time consuming. Moreover, IFC systems are typically more expensive and resource-intensive than conventional flow cytometers, making them less accessible. Despite the most recently proposed solutions have almost reached the goal of finding a balance among volumetric imaging with high resolution, throughput, sensitivity and easiness of operation, they still lack an integration of all the components needed for complex illumination and sample delivery. Device integration is desirable to ensure measurements repeatability over long operation periods, a fundamental step toward a standardization of IFC protocols^10^. One possible solution to the complexity of IFC is the development of integrated, miniaturized platforms encompassing all the optical elements and the fluidic delivery ensuring automated, stable and reproducible operation. Such systems represent significant advancements in flow cytometry technology, offering compact, portable, and versatile platforms for high-resolution, repeatable cellular analysis. Volumetric imaging has been demonstrated by integrating microfluidic lab on chip and microscopy techniques as phase tomography^11,12^, light-sheet fluorescence microscopy^13–21^. Resolution can be also enhanced by implementing advanced techniques as confocal microscopy^22^, light field microscopy^23^ or localization microscopy^24^. We recently proposed the use of an engineered illumination pattern realized by a photonic reconfigurable integrated circuit^25^. In the device light sheet fluorescence microscopy and structured illumination microscopy were combined by exploiting optical fibers and integrated optofluidic devices to achieve high resolution imaging flow cytometry on chip. Here we present an optofluidic platform fabricated by femtosecond laser irradiation followed by chemical etching (FLICE)^26^, composed by an aluminoborosilcate glass chip including reconfigurable integrated photonic circuits for patterned light generation bonded to a fused silica glass chip encompassing cylindrical hollow lenses perfectly aligned to a properly shaped microchannel where the sample under investigation flow. With respect to our previous work^25^ in which we used an array of optical fibers between the two glass substrates, here we have removed that component which inevitably affects the stability of the device performances mainly due to possible and asymmetric mechanical vibrations of the fibers. In this work instead of optical fibers, integrated bended optical waveguides have been used. We also integrated lenses directly into the system to reduce the number of elements requiring alignment and assembly. Nevertheless, these fundamental substitutions have highly increased the complexity of the fabrication process on different levels from waveguides quality to etching uniformity and assembling process complexity, all aspects that have been thoroughly addressed and are here discussed. While this new design introduced certain fabrication challenges, we successfully validated its feasibility by imaging HeLa cells.

## Working principle

Structured illumination microscopy (SIM) uses known illumination patterns to extract information about the fluorophore distribution, achieving a resolution up to two times finer than the optical system’s diffraction limit. Using a known high frequency illumination pattern as excitation and measuring the coarser fluorescence emission pattern (with a minimum feature size larger than the standard resolution limit) it is possible to reconstruct the fluorescence distributions at super-resolution level. From a technological point of view, SIM can be easily integrated in a standard epifluorescence microscope by the use of a diffraction grating in the illumination path^27^, or by using a spatial light modulator to recreate the desired pattern in the image plane of the system^28^. Furthermore, SIM typically exploits spatial light modulators to generate patterns with high speed, allowing temporal resolutions not available with other superresolution techniques as single-molecule localization microscopy, like photoactivated localization microscopy (PALM) or stochastic optical reconstruction microscopy (STORM), opening the possibility to exploit this technique in automatic, high-throughput measurements^29^. Therefore, as in our previous work^25^, here we exploit the interference of two beams propagating at an angle one with respect to the other to achieve structured illumination on chip. The choice of the angle determines the modulation frequency and consequently the maximum obtainable resolution enhancement.

**Fig. 1:**
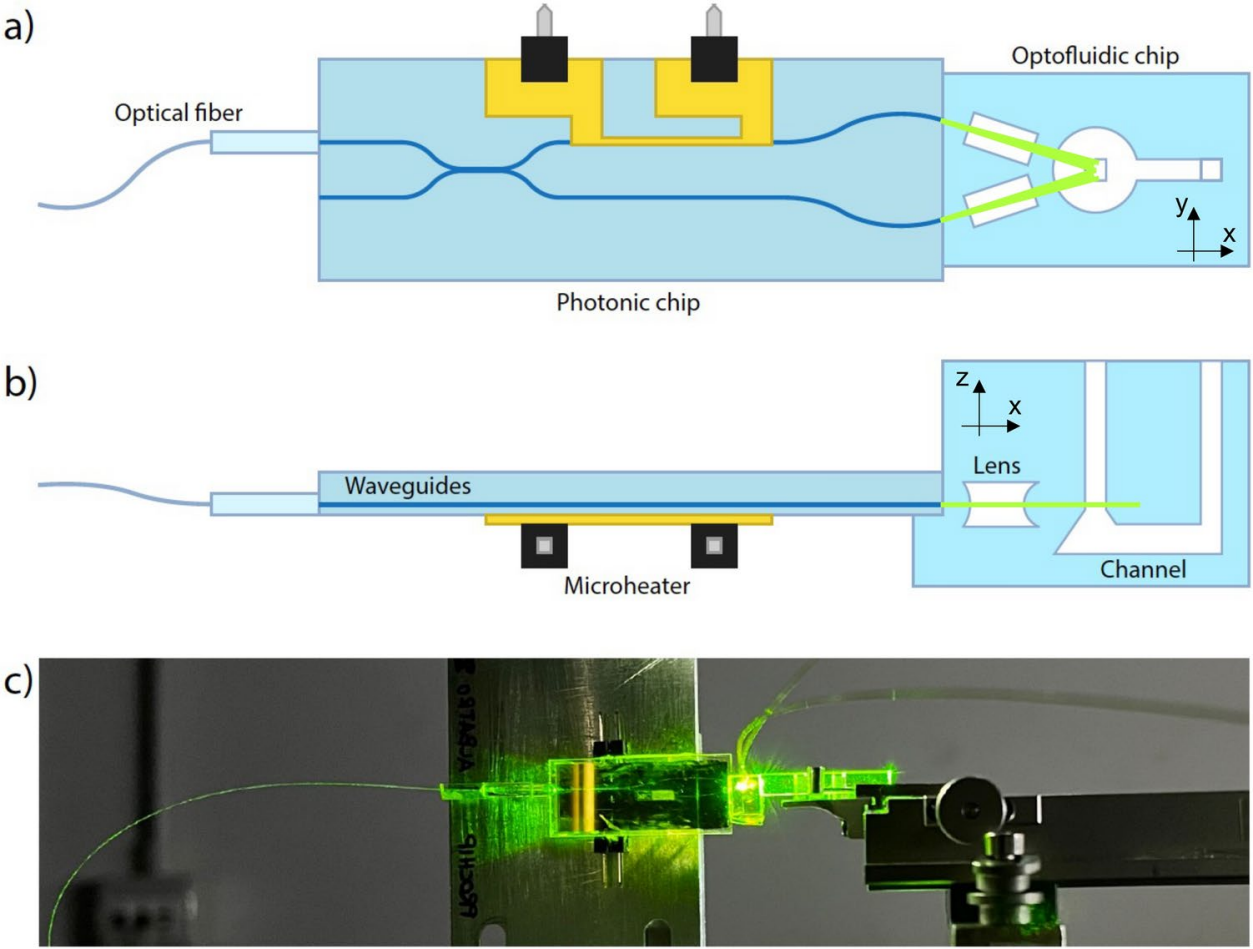
Integrated optofluidic platform design. Two separate glass chips are bonded to obtain a heterogeneously integrated platform. The propagation of the two beams generating the interference pattern in the channel is also shown. (**a**) Bottom view, (**b**) side view and (**c**) photograph of the final device.

Figure 1 shows the schematic of the optofluidic platform composed by an aluminoborosilcate glass photonic chip bonded to a fused silica glass optofluidic chip. Both components of the heterogeneously integrated device have been realized by femtosecond laser micromachining (FLM)^26,30^, a fabrication technique that allows inducing different modifications in transparent substrates as a function of the laser irradiation parameters. Indeed, by tuning the fabrication parameters, material ablation, local refractive index modification and etching selectivity enhancement can be obtained, respectively on the surface and in the bulk of glass substrates, thus leading, in the second case, to the realization of optical waveguides and buried microchannel (after the exposure of the irradiated material to acid or basic etchants) with just one versatile fabrication technique and with a single optical setup. In particular, the photonic chip incorporates an angled beam splitter realized with a directional coupler followed by a thermal shifter, i.e. a gold microresistor, which allows changing the phase of one of the two output arms. The two waveguides at the output of the chip act as two point sources generating the desired interference pattern that can be translated by the controlled phase variation. The two output waveguides have an angle and a distance designed to perfectly match the position of the two air lenses on the second device, once the two components are bonded. The lenses are hollow cavities realized by femtosecond laser irradiation followed by chemical etching (FLICE) designed to focus the output of the waveguides in one direction and oriented in such a way that two light sheets are generated and overlaps in correspondence of the microfluidic channel (also fabricated by FLICE) where the sample flows. The hybrid approach has been chosen as it introduces modularity to the system, which allows exploiting the optimized performances of the two glasses and to easily modify or add components with new functionalities to the system. Optical waveguides with high performances are routinely fabricated in borosilicate glasses. Irradiation followed by etching has been deeply studied and optimized in fused silica by engineering the nanograting formation that allows realizing complex 3D buried hollow channels for the fluidic network, while channel formation process in borosilicate is still under investigation^31,32^. Moreover, the hybrid integration allows introducing the annealing process of the fluidic chip without affecting the optical circuit performances.

## Results and discussion

### Optofluidic component

**Fig. 2:**
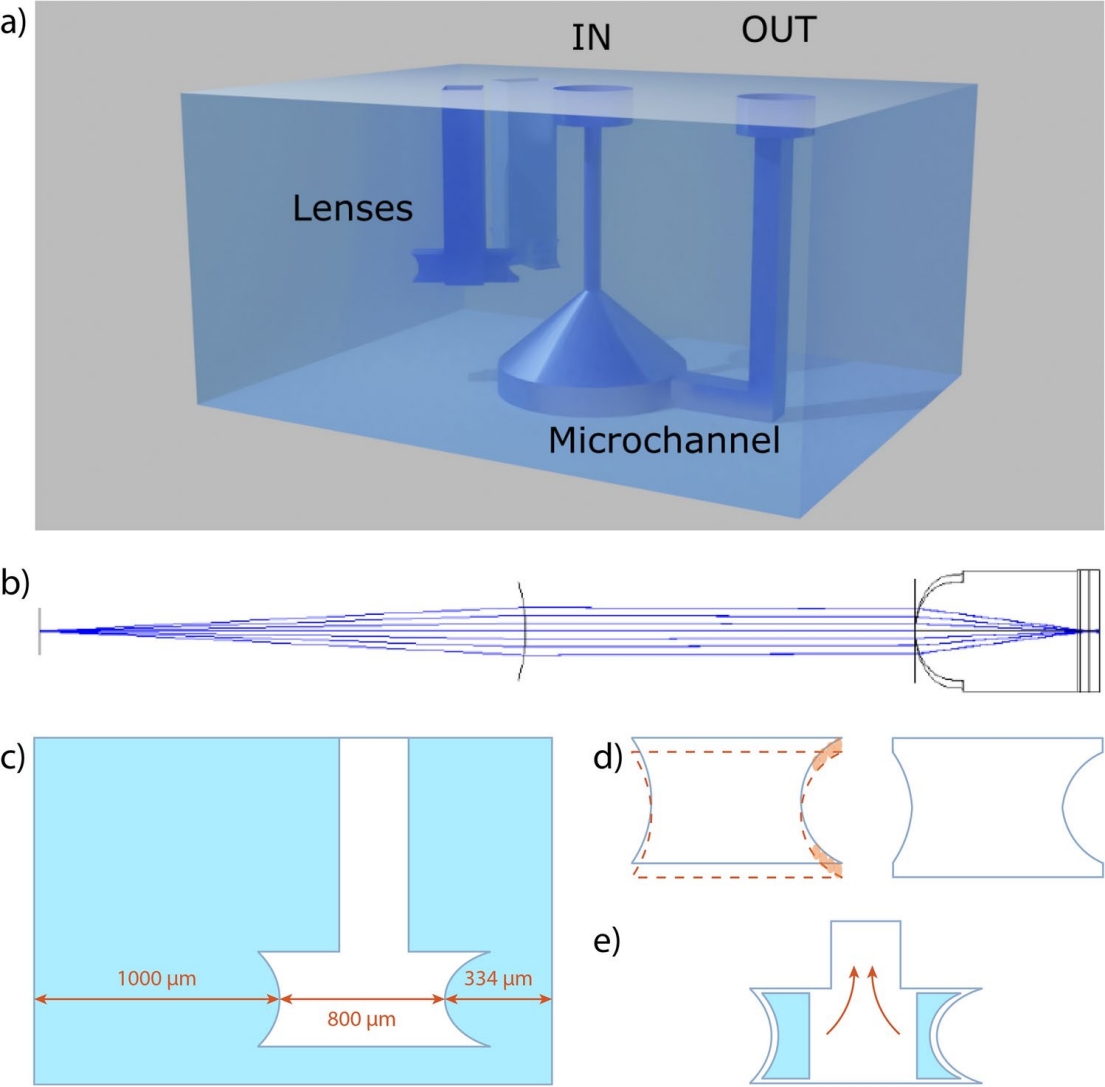
(**a**) 3D rendering of the optofluidic component created with Sketchup (www.sketchup.com). (**b**) Zemax ray tracing scheme exploited for the lens profile design. (**c**) Schematic of the hollow microlenses. The shortest distance corresponds to the distance between the lens and the microchannel, as theoretically evaluated from the simulations of the lens behaviour. (**d**) Compensation of the voxel ellipticity: scheme of irradiated profile for the compensation (left) and resulting etched profile (right). (**e**) Scheme of the inner part ejection to form the empty long cavity lens.

Figure 2a reports a scheme of the optofluidic chip, composed of a microchannel and two lenses. The geometry is similar to that reported in^14^ the main differences being: (i) the cross-section of the detection channel is now pentagonal, see Fig. 1a, as the two light sheets are supposed to enter orthogonally to the channel wall; (ii) the lenses have been designed with a long cavity to mitigate possible Fabry–Perot effect inside the air cavity.

We carried out the lens profile optimization using optic studio (Zemax) to simulate with few modifications a lens geometry already discussed in^14^, firstly with a ray tracing approach, and then with a physical optics propagation module, as schematically shown in Fig. 2b, with the aim of obtaining the light sheet thickness as uniform as possible within the microchannel section. Such optimized lens profile gives a FWHM of $3.4~\upmu {\rm m}$, $1.1~\upmu {\rm m}$ and $3.2~\upmu {\rm m}$ respectively at $-20~\upmu {\rm m}$, $0~\upmu {\rm m}$ and $+20~\upmu {\rm m}$ from the center of the channel. The optofluidic chip geometry has an important challenge in its fabrication: the two lenses are not orthogonal one to each other, thus they can’t be both realized exploiting a longitudinal configuration as irradiation scheme, which would allow the highest accuracy in the pattern irradiation and the lowest roughness, as described in^14,33^. The lenses have then been irradiated in a transversal configuration, i.e. the irradiation beam direction is perpendicular to the focusing plane of the lens. For this reason, the effect of the fabrication voxel ellipticity (elongated along the beam propagation direction) becomes relevant for the design, and the irradiated lens profile can strongly differ from the theoretical one. We therefore decided to exploit for the irradiation a 40× 0.75 NA water immersion (WI) objective, to reduce the laser spot dimension and at the same time the spherical aberrations due to the refractive index mismatch between the glass substrate and the surrounding air. After irradiation, the device was etched in an HF acid aqueous solution at 20% volume concentration at $35^{\circ }{\rm C}$ in an ultrasound bath. Recursive optimization of the irradiation and etching process has been performed to calibrate and compensate for residual elongation of the focal volume and achieving a lens profile matching the theoretical one, as schematically shown in Fig. 2c,d. All the structures have been realized to etch only the outer surface of the channels and remove the inner part by ultrasound vibrations, as depicted in Fig. 2e for the case of the lens cavity.

**Fig. 3:**
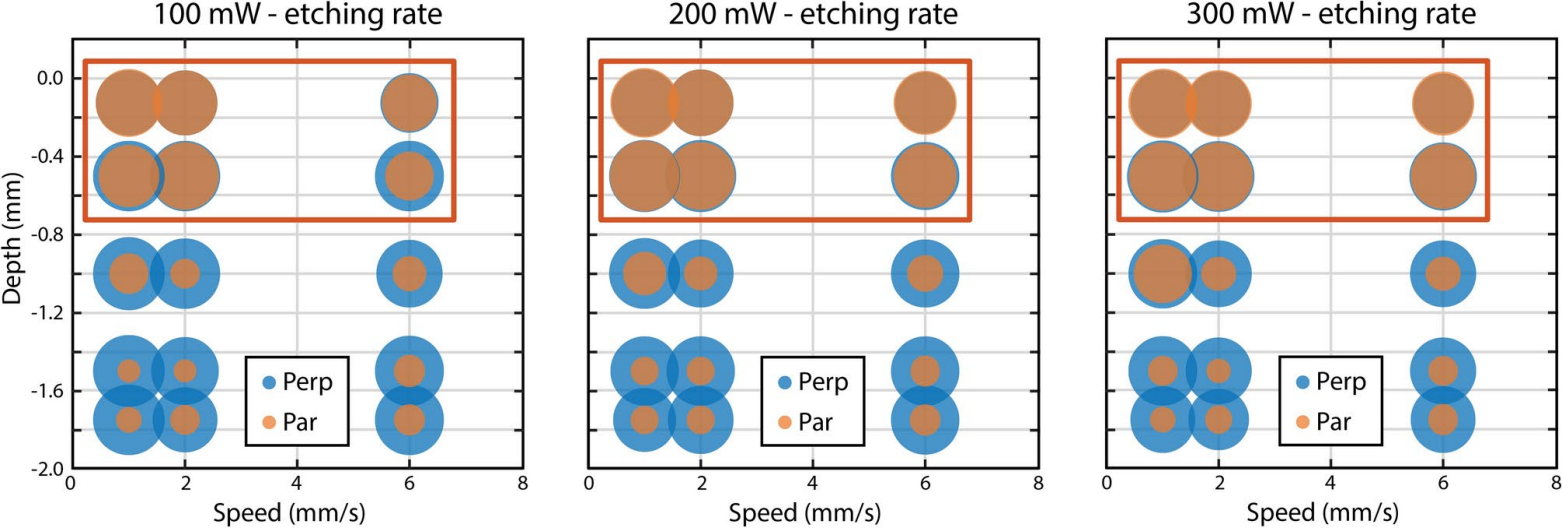
Results of the irradiation parameter optimization to achieve enhanced etching selectivity with a 40x, 0.75NA WI objective. The graphs show the normalized etching rate, as a function of the irradiation parameters (power, irradiation depth, translation speed) both for the laser beam polarization directions parallel (orange) and perpendicular (blue) to the beam scanning direction. The radius of the circles is proportional to the etching rate, as measured after 30 min of etching in hydrofluoric-acid solution. The results allowed the identification, highlighted by the red squares, of a fabrication window showing an optimized isotropic etching independent from the beam polarization orientation respect to the scanning beam direction at fabrication depths up to 0.6 mm.

A second issue deriving from the lens geometry is the different etching rate selectivity related to the nanograting formation, whose orientation depends on the beam polarization with respect to the beam scanning direction^34^. The highest etching rate is typically achieved when the beam polarization direction is perpendicular to the beam scanning direction, configuration that can not be applied in our irradiation scheme, unless the beam polarization is varied during the fabrication or circular polarization is exploited, typically resulting in a lower etching rate. The chosen WI objective is not typically used for microchannel fabrication therefore we first performed an optimization of the fabrication parameters by irradiating the fused silica substrates with the second harmonic of a commercial femtosecond laser source at 1040 nm wavelength, 1 MHz repetition rate and pulse energy up to 23 $\upmu$J (femtoREGEN, High Q Laser). The optimization was performed by varying both the irradiation power and the scanning velocity at different depths for polarization parallel and perpendicular to the scanning direction. The results of the optimization are reported in Fig. 3, which shows the etching rate for both polarization cases as a function of the scanning velocity and the irradiation depth for three different energy values. The plotted values are the etching rates achieved after 30 min etching in a 20% volume concentration at $35^{\circ }{\rm C}$ in an ultrasound bath, normalized to the highest obtained value. The search for the optimal parameters for enhanced etching selectivity allowed the identification of a fabrication window that guarantees an isotropic etching rate up to a fabrication depth of 0.6 mm, defined by a pulse energy in the interval $100 - 300\,{\rm nJ}$ and a translation speed of $0.5 - 2.5\ {\rm mm/s}$. In this window, the etching rate of the tracks written with parallel polarization is always between 75% and 85% of the perpendicular one, which is typically the preferential one, and is found to be equal to $1400~\upmu {\rm m}/{\rm h}$ for an irradiation energy of 300 nJ, a scanning speed of 1 mm/s at $500~\upmu {\rm m}$ depth. These findings allowed overcoming the necessity to rotate the polarization or to exploit circular polarization to have a uniform etching rate. A regime of isotropic etching was also found by Ochoa et al.,^35^, by using a lower NA aperture objective; anyway, as already mentioned we preferred in this case a high NA objective to keep the voxel dimension rounder for the lens profile fabrication. We believe that this results significantly increases FLM capabilities, opening to the fabrication of more complex device designs.

### Photonic device

**Fig. 4:**
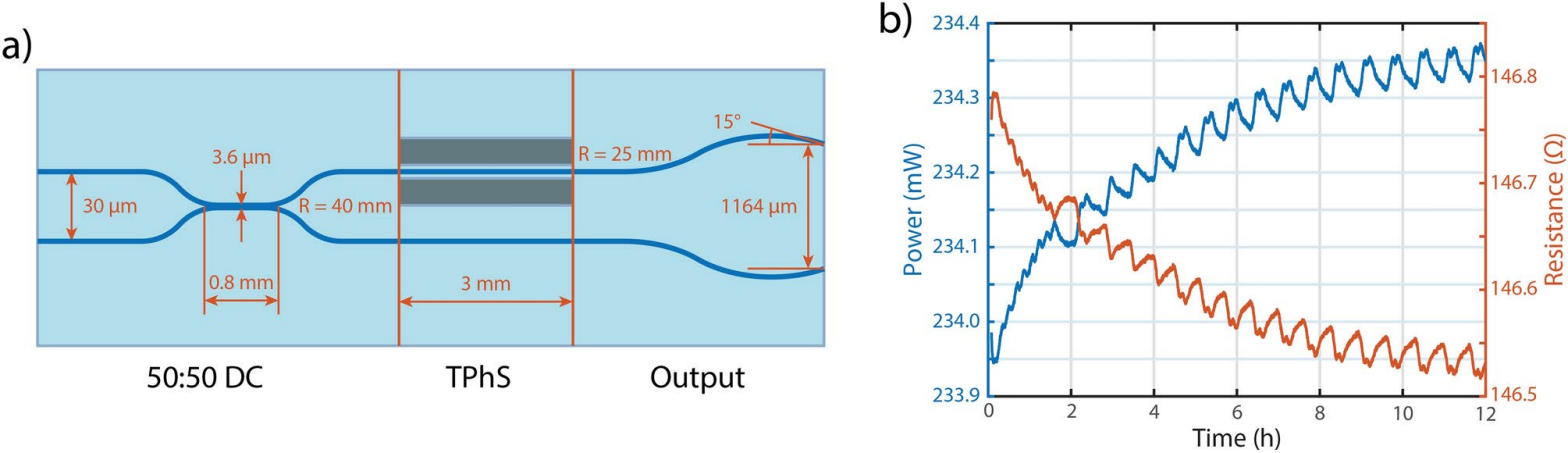
(**a**) Photonic circuit design and feature dimensions. The circuit is divided into three sections: beam splitter (50:50 DC), thermal shifter (TPhS) and angled output waveguides (output). (**b**) Thermal shifter stability over time ($V =$ 5.86 V), showing a peak-to-peak variation of 0.18% over 12 h of continuous operation. The initial warm up transient (5 min) has been removed for graphic purposes. The faint slow oscillations modulating the curves are attributed to the imperfect temperature control of the device ($\Delta T \simeq \pm$0.03 °C).

We fabricated the photonic device from alumino-borosilicate Eagle XG glass, optimizing it for operation at a 561 nm wavelength. The waveguides were written with a 50× 0.65 NA objective with aberrations compensation collar, using an Yb:KYW cavity-dumped mode-locked laser oscillator, with emission wavelength of 1030 nm, pulse duration of 300 fs, and a repetition rate of 1 MHz. To obtain single-mode waveguides we exploited, as described elsewhere, a multiscan approach followed by thermal annealing^36^. In detail, the waveguides have been irradiated at a distance of 15 $\upmu {\rm m}$ from the bottom surface of the glass substrate with 230 mW of laser power, 40 mm/s of scan velocity and 8 overlapped irradiations. We have obtained single-mode waveguides with propagation losses of about 0.3 dB/cm and symmetric mode dimensions (FWHM) of about 2.7$\times$2.8 $\upmu {\rm m}^2$ at 561 nm. The photonic circuit includes three sections: a 50:50 beam splitter implemented with a directional coupler (DC), the thermal shifter section (TPhS) and the angled outputs. The geometrical parameters are reported in Fig. 4. To fit with the optofluidic component and to obtain the designed SIM frequency pattern, the two waveguide outputs should be placed at a distance of 1.164 mm with an angle of $15^{\circ }$. This aspect is tricky, as we need to irradiate the waveguides and polish the end facet of the chip to optical quality keeping a perfect alignment after bonding between the photonic and fluidic components. To guarantee perfect positioning of the outputs we tried to close the shutter while the irradiation beam is still focusing inside the glass, anyway this approach led either to a tapering or to a damage of the waveguides. We therefore decided to engrave by laser irradiation a mark on the glass surface in correspondence of the ideal final position of the waveguides with an ablation line and we then polished the facet till that position.

According to the simulation results reported in^37^, shallow trenches around the thermal shifter section, with a geometry of $5~\upmu {\rm m}$ width, 8 and $16~\upmu {\rm m}$ depth and $15~\upmu {\rm m}$ distance from the waveguide, allow achieving a fast switching time with the thermal shifter of about 1 ms. Differently than in our previously reported devices here we decided to facilitate and fasten the photonic chip fabrication by realizing the trenches in the same irradiation step of the waveguides, exploiting the same objective with the following parameters: 300 mW as optical power and 0.1 mm/s as writing speed. This guarantees a perfect alignment between the two components. From the geometrical point of view, we irradiated two adjacent lines ($1~\upmu {\rm m}$ spacing) per horizontal plane and we used a vertical spacing of $0.5~\upmu {\rm m}$ for the first $5~\upmu {\rm m}$ and a spacing of $1~\upmu {\rm m}$ afterwards, writing in bottom to top approach.

The thermal shifter was fabricated according to the process reported in^38^. After a piranha cleaning, a Cr/Au (5/100 nm) film was deposited on the substrate with a magnetron sputtering process (Leybold LH Z400) and then a thermal annealing step (400 °C for 1 h) was employed to stabilize the film and avoid long term drifts of the resistance value (and thus of the induced phase) during the operation. Last, patterning of microheater and contact pads was done with the same femtosecond laser used for the waveguide/trench inscription (10×, 0.25 NA focusing objective, 200 mW of average laser power and 2 mm/s scan velocity). In order to verify that the high mechanical stability reached through the integration process of this platform was not spoiled by electrical drifts, we measured the resistance (and power dissipation) over time for a constant applied voltage $V =$ 5.86 V, which is the maximum operating voltage for the system (see "Integrated platform assembly and characterization" section). Both voltage supply and current sensing were performed at the same time through a Keysight B2902A source/measurement unit, while the device was mounted on a Peltier cell in order to stabilize the temperature through a closed-loop controller. The result was a remarkable electrical stability, with a peak-to-peak variation as low as 0.18% measured over 12 h of continuous operation (Fig. 4b). Since phase depends linearly on power dissipation, this value represents also the electrical contribution to the overall phase stability of the device.

### Integrated platform assembly and characterization

After the glass devices realization, the two devices have been separately finalized and then assembled together. Microfluidics assembling mainly consisted in device sealing and tube connection by means of PEEK capillaries (IDEX). In both cases the sealing has been performed using UV curable glue (photobond GB345, DELO) applied manually on the site, using capillary force driven diffusion of the glue film to coat the desired surface. The same glue has been used for the optical fiber pigtailing of the photonic device to a polarization maintaining optical fiber. Afterwards, it has been glued on a custom holder to keep the photonic chip upside-down to favor the alignment between the waveguide outputs and the lenses within the fluidic chip. In order to ease the alignment, laser ablated marks have been inscribed on the two devices during the fabrication process. The superpositions of these marks in the assembling phase guarantee the alignment of the waveguides output with the lenses optical axis along the horizontal plane of the device. To check the alignment, the channel is filled with rhodamine and the light sheet shape is real-time monitored with a camera. An image of the device during the assembling is reported in Fig. 1. After the assembling, a heat sink has been glued on the top of the photonic chip, using a thermal conductive adhesive tape.

**Fig. 5:**
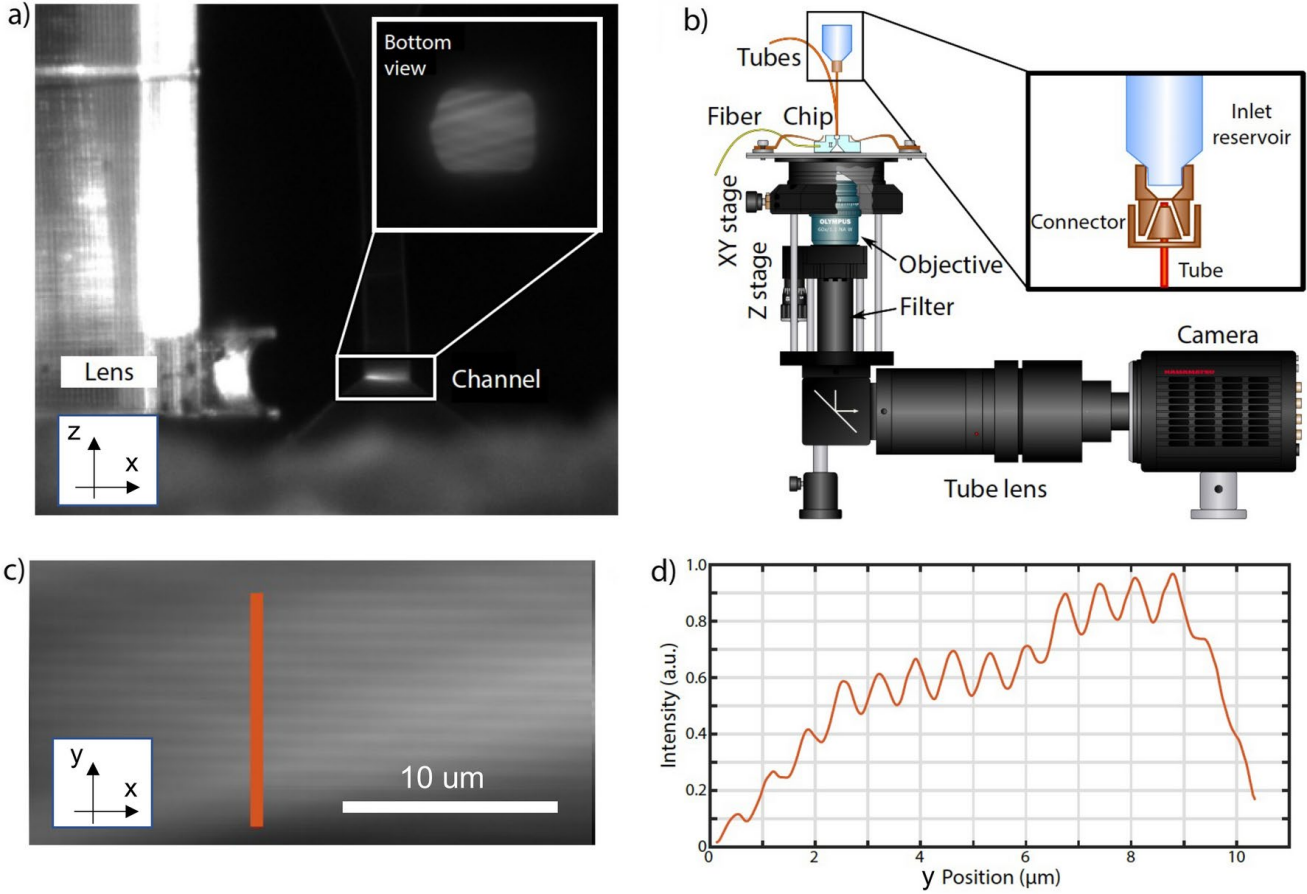
(**a**) Side-view picture of the device during the assembling procedure. The fluorescence profile of the light-sheet generated by the rhodamine dye is visible in the channel. In the inset is reported the bottom view of the light-sheet during the assembling procedure. This view shows a non perfectly uniform illumination. (**b**) Schematic of the setup used for the pattern visualization inside the microfluidic channel. (**c**) ROI of the cross section (bottom view of the light sheet in the channel) picture acquired with a high NA objective. Even if the illumination is not uniform, the modulation given by the interference is clearly visible. The scalebar is 10 $\upmu {\rm m}$. (**d**) The graph reports the intensity (in gray levels of the camera) along the red line indicated in panel (**c**)..

The setup used for the chip bonding was a high-precision dedicated workstation, consisting in a 4 degree of freedom sample holder and 2 motorized positioners (Miniature Hexapodes, H-811.F2, PI) with 6 degrees of freedom. This setup was equipped with up to three cameras (CCD camera, Thorlabs) and the possibility to insert different microscope objectives and filters, to monitor the chips alignment through the fluorescence emitted signal. Figure 5a shows a side-view picture of the chip during the alignment and bonding procedure. The profile of the cylindrical lens can be appreciated as well as the fluorescence emitted by rhodamine excited by the light sheets intercepting the michrochannel. In the inset the cross section of the channel is also reported by showing the bottom view of the fluorescence signal emitted by the rhodamine solution. Figure 5b reports instead a schematic of the detection path, in particular the chip was aligned on a Nikon microscope (TU2000) equipped with a 40x WI objective and a 1.5× tube lens. A 561 nm OBIS laser (Coherent) was coupled to the chip and the generated pattern was imaged through a high speed CMOS camera (Hamamatsu Orca Flash 4.0 V3). The interference pattern has been characterized by looking at the fluorescence emitted by a rhodamine solution filling the channel, as shown in Fig. 5c,d, where an enlargement of a region of interest (ROI) of the bottom view of the rhodamine fluorescence signal, excited by the interfering light sheets, is reported. The uniformity of the light sheet is limited by the roughness of the etched walls of the cylindrical lenses. Indeed, even if the irradiation geometry, having the surfaces of interest parallel to the irradiation beam propagation direction, is allowing low roughness, as described in^25,33^ and references therein, we expect a value around 60–100 nm for the lens. This value could be improved by exploiting polishing methods that act in the bulk of a substrate and not only on its surface. We performed a scan of the thermal shifter driving voltage, to obtain the curve representing the relation between V and the applied phase shift (read as a pattern rigid shift). A custom made Scopefoundry software was exploited to acquire 11 frames per set, corresponding to 11 driving voltage steps of 0.5 V each from 2 to 7.5 V. We acquired each set 8 times, obtaining a dataset with 11 × 8 images. The acquisition frequency was set to 100 Hz over a 512 px by 700 px region of interest. The images collected in this way were analyzed with a combination of ImageJ and custom MATLAB software. As a first step each frame (corresponding to a single voltage step) had been averaged across the 8 acquisitions (using z stack average functionality of ImageJ). Afterwards a line profile was taken in a region with uniform light sheet. The MATLAB software high-passes the signal to remove the light sheet modulation, without filtering the pattern fringes. Then it detects the peaks and compute the shift between each voltage step averaging over more than 20 peaks. In this way a geometrical shift is obtained for each voltage input, together with an average period of the modulation. These two data are used to obtain the phase shift ($\Delta x / period$). The obtained pattern period was 791 nm, which is in good agreement with the theoretical one of 754 nm, obtained with a semi-angle of $15^{\circ }$ between the two interfering beams. According to SIM theory three images at phase shifts of 0, $2/3 \pi$ and $4/3 \pi$ are needed; these values could be achieved in our characterization by applying voltages of 0 V, 4.15 V and 5.86 V respectively.

### Biological validation

We have experimentally validated our heterogeneously integrated platform by imaging cells from a fixed population of HeLa cells fluorescently marked using Wheat Germ Agglutinin Alexa Fluor 594 (WGA 594) conjugate to label plasma membranes, diluted in a liquid suspension of PBS.

**Fig. 6:**
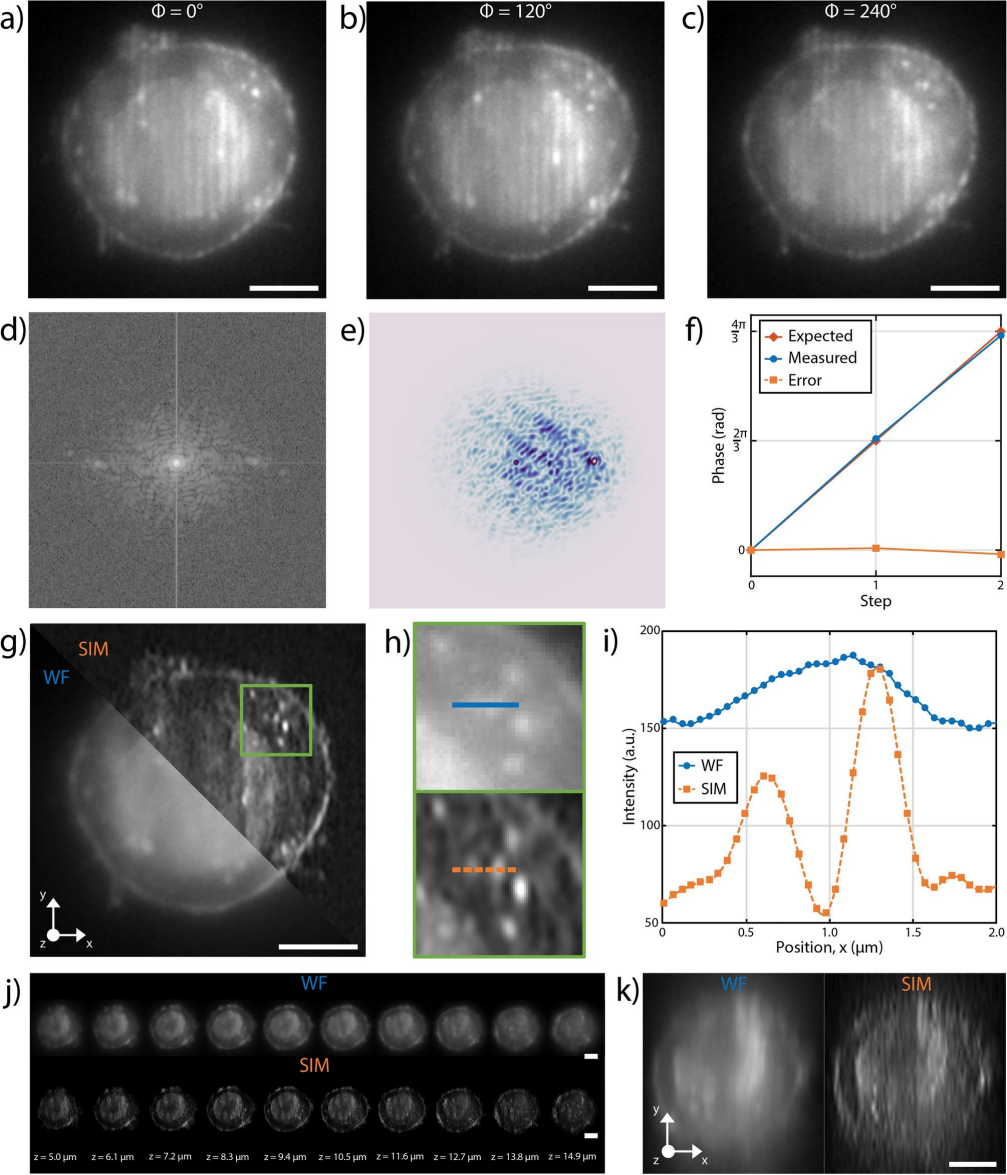
Acquisition of a fixed Hela cell labelled with WGA 594 immersed in water during its flow through the patterned light sheet. (**a**–**c**) Raw images of a single plane for different phase shifts. (**d**) Fourier transform of the acquired fluorescence signal. (**e**) Cross-correlation of the image in frequency space. (**f**) Comparison between the expected and measured phases that were applied. (**g**) Widefield (WF) and reconstructed SIM image of the plane shown in **a**–**c**. (**h**) Detail of (**g**) highlighted by the green box, showing the resolution improvement after SIM reconstruction (bottom panel) with respect to the WF (top panel). (**i**) Intensity profiles retrieved along the horizontal lines (x direction) shown in (**h**), highlighting the improvement obtained both in resolution and contrast with the SIM reconstruction. (**j**) Selected single planes, equally spaced of 1.1 $\upmu {\rm m}$, acquired during the cell flow through the patterned light-sheet in WF (top panel) and SIM (bottom panel) modality. (**k**) View of the x-z plane of the cell acquired in (**j**), showing the improvement of the reconstructed SIM (right panel) with respect to the WF case (left panel). Scale bar is always 5 $\upmu {\rm m}$.

High-precision pressure-driven pumps have been used to process the sample. Cells were flown at about 10 $\upmu {\rm m/s}$ and the image acquisition was performed at 50 Hz, with a 40×, 1.15 NA water immersion Nikon objective and tube lens with an additional magnification of 1.5×. The operation of the system at 13 nl/min allows the acquisition of 40 cells/min, each sectioned with 15 planes. The throughput could be further increased by adjusting the acquisition rate (up to 400 Hz) and the sample concentration. Commercial imaging flow cytometers can reach throughput of up to 5000 cells/sec, a value far from our system performances. Anyway, these instruments allow only two-dimensional analysis at standard/low resolution, therefore the high throughput capability of our system is hindered in the high-resolution volumetric imaging. Image acquisition is performed with a custom software developed in Python that synchronizes the camera acquisition with the thermal phase shifters and with the laser. A Python module (napari-sim-processor) is used for enhanced resolution image reconstruction. The results are reported in Fig. 6. In particular in Fig. 6a–c we present the images of a single plane showing the patterned light-sheet with the 3 phases. As a measure of the quality of the applied phases we have retrieved the image in the frequency space. However, it is complex to determine with high precision the frequency of the carrier. To this purpose, we use the method based on cross-correlation shown in^39^, which allows us to retrieve the carrier frequency. A metric of the quality of the applied phases is given by the reconstruction of the phases themselves, which are compared to the expected ones as shown in Fig. 6f). SIM images can then be reconstructed (Fig. 6g–k). An average improvement of 1.33 in the resolution in one dimension has been obtained with respect to the widefield image, as retrieved by the Fourier analysis. A few examples showing the resolution improvement are visible in Supplementary Fig. S1. Moreover, an overall improvement of the image quality is visible and is obtained by the application of the retrieval algorithm, which results in further background reduction and contrast enhancement. The enhancement in image quality and resolution is also shown in the detailed analysis of the single plane of various acquired images of vesicles from fixed HeLa cells shown in Supplementary Fig. S2. Finally, it is possible to acquire plane by plane the samples (Fig.6j) and reconstruct it in 3D. Figure 6k shows a x-z plane showing the stability of the flow, which does not require registration of the acquired planes and further shows the imaging improvement. The 3D reconstruction of the cell is reported in Supplementary Fig. S3, along with the analysis of a second sample that is shown in Supplementary Fig. S4.

## Conclusion

In summary, we developed a heterogeneously integrated optofluidic platform that performs high-resolution imaging flow cytometry. This was achieved by implementing a combination of light sheet and structured illumination fluorescence microscopy in a microfluidic environment. Advanced laser manufacturing allowed the extreme integration of all the optic and fluidic components thanks to its versatility and its precision in the fabrication of the components and in favouring the alignment and bonding of the two glass chips. The high level of integration raised several challenges in the fabrication of both the photonic and the optofluidic chip. The first has been solved by exploiting an already known glass platform, i.e. borosilicate glass, and refining the reconfigurable circuit fabrication that was previously optimized^37^. The second was resolved by optimizing the irradiation and etching process with a WI objective. This allowed us to explore new regimes where the etching rate became isotropic, losing its well-known dependence on the laser’s polarization direction relative to the scanning direction. The realization of a fully integrated platform avoiding the use of any moving parts and also of optical fibers allowed obtaining a platform with long-term stability towards both mechanical and thermal stresses from the environment. The platform layout has been here presented and its functioning has been validated by imaging healthy HeLa cells labelled with Alexa fluorescent marker. The chip performances could be further improved by optimizing the light sheet uniformity and the sectioning capabilities, which would both benefit by the use either of thermal annealing (still in optimization) or plasma polishing procedures (future perspectives) to decrease the residual roughness of the embedded lenses surfaces after the irradiation and etching process. Nevertheless the promising results achieved in the bio validation open the way to the use of such a device for large cell population phenotyping or drugs screening for personalized therapies efficacy evaluation. This can be achieved benefiting from the measurement automation and the long term mechanical stability ensured by the full integration of the components that ensures stable alignment over long time operation, as well as the portability of the device for in situ operation by non specially trained personnel.

## Supplementary Information

Supplementary Information.

## Data Availability

All data generated or analysed during this study are included in this published article
