# Supplementary material for: Femtosecond laser microfabrication of a fully-integrated optofluidic device for 3D imaging flow cytometry
Source: Sci Rep. 2025 Apr 8;15:11950. doi: 10.1038/s41598-025-93118-x (PMC11978988; doi:10.1038/s41598-025-93118-x)
Supplement: Supplementary file 1 — Supplementary Information. [file 41598_2025_93118_MOESM1_ESM.pdf]

Supplementary Material

Femtosecond laser microfabrication of a fully-integrated optofluidic device for 3D imaging flow cytometry

Federico Sala[1], Petra Paiè[2,1], Alessia Candeo[2,1], Francesco Ceccarelli[1], Roberto Osellame[1], Andrea Bassi[2,1] and Francesca Bragheri[1*]

[1]Istituto di Fotonica e Nanotecnologie, Consiglio Nazionale delle Ricerche, Piazza Leonardo da Vinci, 32, Milano, 20133, Italy.
[2]Dipartimento di Fisica, Politecnico di Milano, Piazza Leonardo da Vinci, 32, Milano, 20133, Italy.

*Corresponding author Email: francesca.bragheri@cnr.it

This file includes:

Figs. S1 to S4

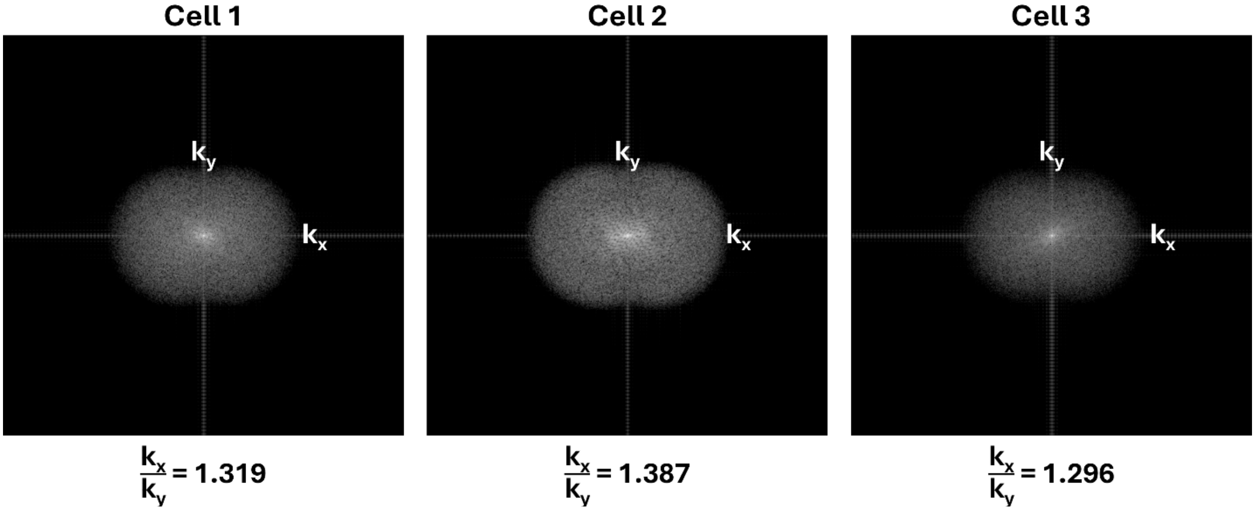

Supplementary Figure S1 Resolution improvement Resolution improvement calculated for 3 different datasets, obtained from the ratio between the highest frequency observable along the x and y axis of the Fourier Transform of the reconstructed SIM dataset. In fact, the y direction is not affected by the improvement in resolution, since the pattern is vertical, while the x direction experiences the maximum improvement.

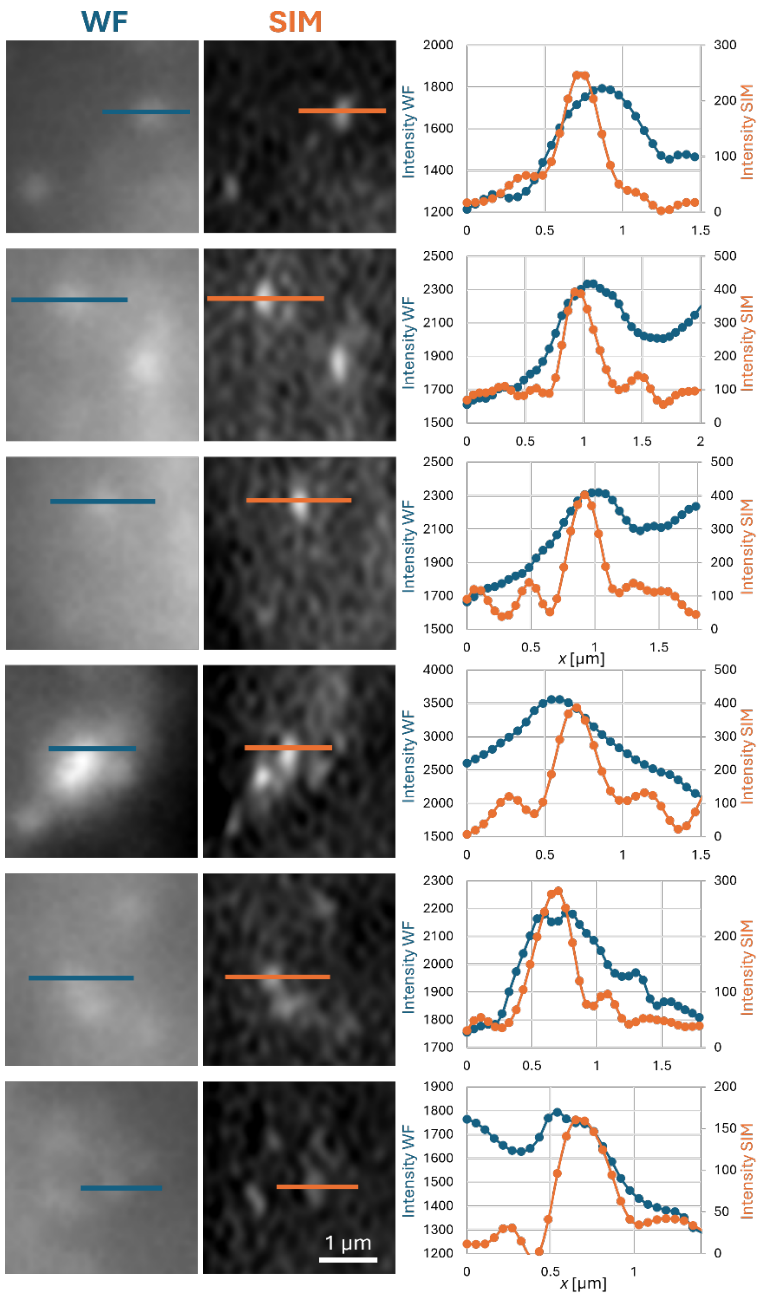

Supplementary Figure S2. Detailed analysis of the resolution enhancement achieved with SIM Details of WF and reconstructed SIM images of one single plane of various acquired images of vesicles from fixed Hela cell labelled with WGA 594, showing the resolution improvement after SIM reconstruction (right panel) with respect to the WF (left panel). Intensity profiles retrieved along the horizontal lines (x direction) shown on the correspondent WF and reconstructed SIM images on the left, highlighting the improvement obtained both in resolution and contrast with the SIM reconstruction.

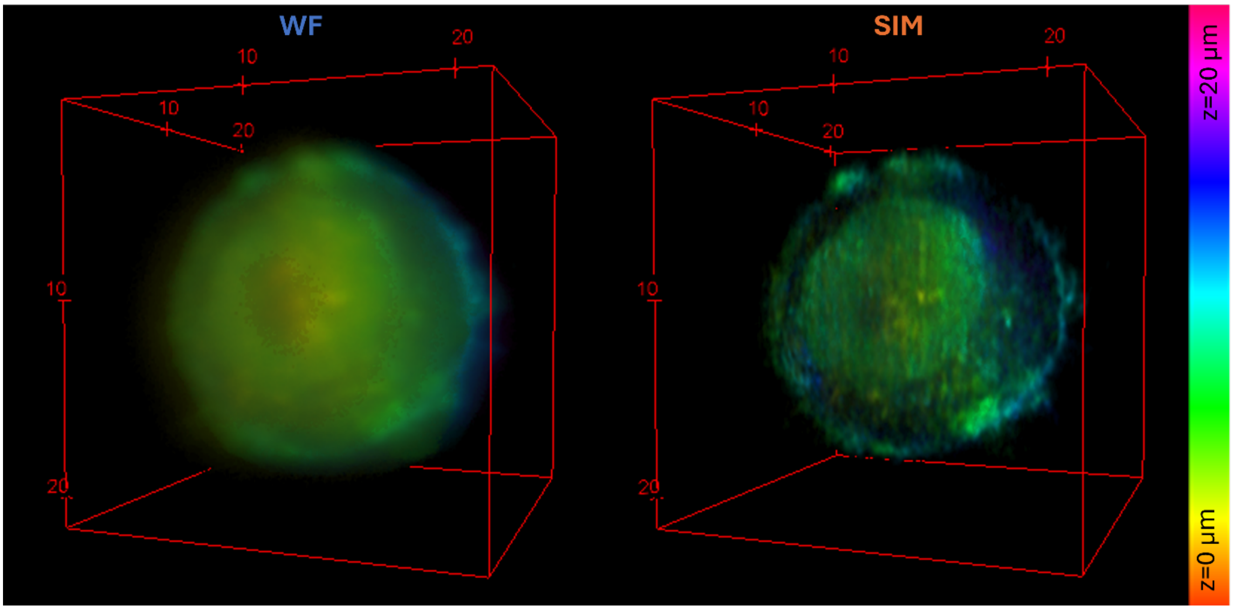

Supplementary Figure S3 3D of the widefield (WF) and of the reconstructed SIM dataset of the cell analysed in Fig. 6. The colors represent the depth, as shown by the colorbar on the side. Scalebar on the 3D box is in micrometers.

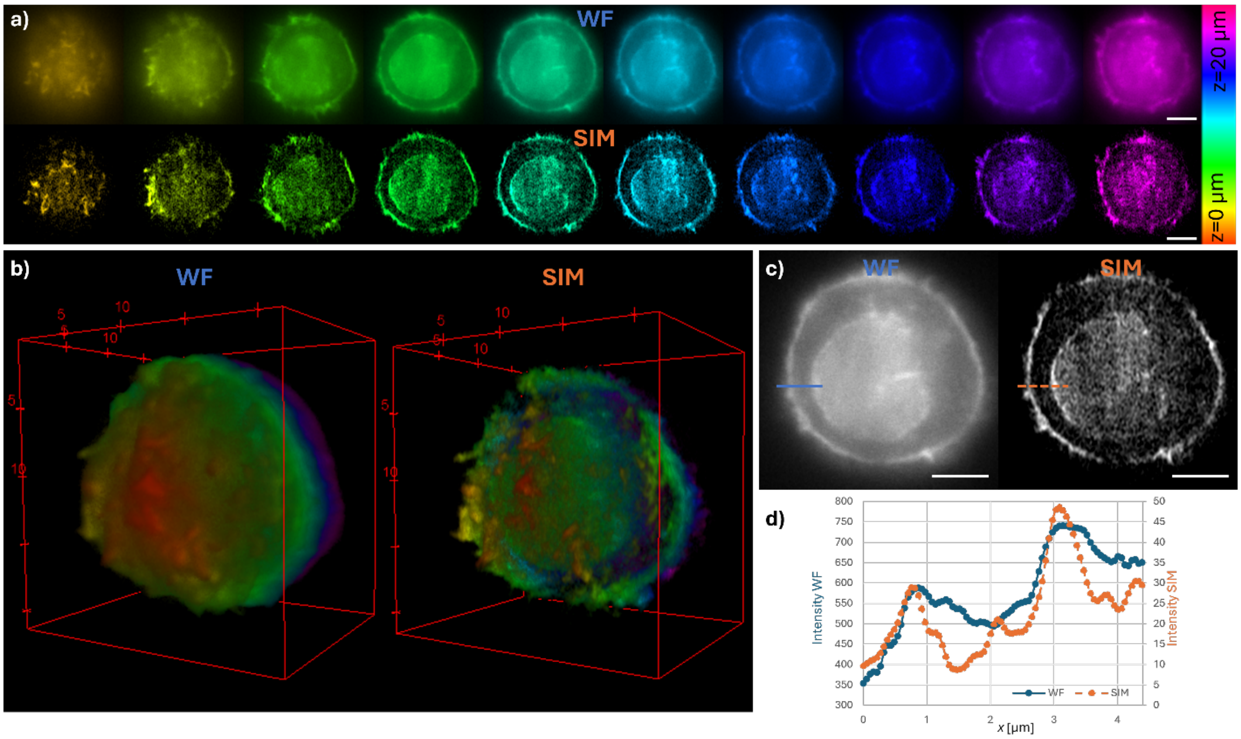

Supplementary Figure S4 Acquisition of a fixed Hela cell labelled with WGA 594 immersed in water during its flow through the patterned light sheet. a) Selected Widefield (WF) and reconstructed SIM single planes of the cell at various depths, equally spaced, with colors representing the depth, as shown by the colorbar on the side. b) 3D reconstructions of the WF and of the reconstructed SIM dataset. The colors represent the depth, as shown by the colorbar of panel (a). Scalebar on the 3D box is in micrometers. c) WF and reconstructed SIM images of one single plane, showing the resolution improvement after SIM reconstruction with respect to the WF. d) Intensity profiles retrieved along the horizontal lines (x direction) shown in c), highlighting the improvement obtained both in resolution and contrast with the SIM reconstruction. Scale bar is always 5 µm.
